# Activated Biocarbons Obtained from Plant Biomass as Adsorbents of Heavy Metal Ions

**DOI:** 10.3390/ma15175856

**Published:** 2022-08-25

**Authors:** Małgorzata Wiśniewska, Magdalena Marciniak, Marlena Gęca, Karolina Herda, Robert Pietrzak, Piotr Nowicki

**Affiliations:** 1 Department of Radiochemistry and Environmental Chemistry, Institute of Chemical Sciences, Faculty of Chemistry, Maria Curie-Sklodowska University in Lublin, M. Curie-Sklodowska Sq. 3, 20-031 Lublin, Poland; 2 Department of Applied Chemistry, Faculty of Chemistry, Adam Mickiewicz University in Poznań, Uniwersytetu Poznańskiego 8, 61-614 Poznan, Poland

**Keywords:** activated biocarbons, chemical activation, waste biomass utilization, heavy metal ions removal, Pb(II) and Cu(II) adsorption, physicochemical and electrokinetic properties

## Abstract

This paper deals with the adsorption of heavy metal ions on the surface of carbonaceous materials obtained via the chemical activation of biomass. Waste plum stones, pine sawdust and horsetail herb were used as the precursors of carbonaceous adsorbents. The effect of the precursor type and preparation procedure on the physicochemical properties of activated biocarbons and their sorption abilities towards Pb(II) and Cu(II) ions have been checked. The obtained micro-mesoporous activated biocarbons were characterized by determination of elemental composition and ash content, the number of surface functional groups and pH of water extracts as well as textural study based on low temperature nitrogen adsorption/desorption and scanning electron microscopy. Additionally, the electrokinetic studies including solid surface charge density and zeta potential determination were performed. Moreover, the adsorption data modelling (equilibrium and kinetics), XPS results analysis and comparison of parameters characterizing electrical double layer formed at the solid-liquid interface enabled the specification of the mechanism of heavy metals binding with the activated biocarbons surface. The maximum adsorption capacity towards copper and lead ions (177.5 and 178.1 mg/g, respectively) was found for plum stone-based activated biocarbon. For all carbonaceous materials, better fit to the experimental data was achieved with a Langmuir isotherm than a Freundlich one. In turn, a better fit of the kinetics data was obtained using the pseudo-second order model.

## 1. Introduction

Environmental pollution and degradation are some of the most serious problems all over the world. New methods of wastewater treatment and recycling should be found in order to limit the impact of human activity on the natural environment. Searching for new, renewable materials useful for industry and science is necessary because of the depleting resources of raw materials. Waste biomass pyrolysis and/or activation are the processes which make all the above possible and have a positive influence on the environment. Decreasing the greenhouse gas emissions as the result of decay process and reducing the amount of stored waste are the result of biomass processing. Bio-oil, biochar and pyrolytic gas are useful products formed during this process [1]. In this way, we can reduce the storage or incineration of such waste as fruit stones, nut shells, sawdust, corn cobs, sunflower husks, straw or herbal herbs [2,3,4].

Biochars (the main products of biomass pyrolysis) are widely used as adsorbents of different inorganic and organic impurities from liquid phase. Additionally, the ease of their activation and modification allows for the significant development of their porous structure and functionalisation of the surface. Metal ions, synthetic dyes, phenols, amines or pharmaceuticals are commonly adsorbed on the biochars, activated biocarbons (products of physical and chemical activation of biomass) or different kinds of carbon-based nanocomposites [5,6,7,8,9,10,11,12,13]. These materials are low-cost adsorbents and they can be easily regenerated. Moreover, due to their natural origin, they are environmentally friendly adsorbents and their disposal is not troublesome.

Lead is one of the most dangerous inorganic pollutants present in water and soil. It is slightly soluble in water, but it is well adsorbed on the surface of soil and sludge, which causes its high availability for living organisms. Lead serves no useful biological function in human body. The metal enters it through ingestion, inhalation or skin contact. The biochemical basis for lead toxicity is its ability to bind the biologically-important molecules, thereby interfering with their function by various mechanisms [14]. Many remediation techniques are being developed for the lead ions extraction from water and soil, however adsorption seems to be the highest effective and economically justified method. Two mechanisms of lead adsorption are possible-ion exchange and complexation [15]. Many adsorbents could be used in this process including biochars and activated biocarbons.

Copper is an essential element for life; however its high concentration can be toxic. It is used in jewellery, medicine, cosmetics and the metallurgical industry. Copper is applied as a feed additive due to its growth-promoting effect [16]. Due to these properties, copper should not get into the environment in an uncontrolled manner. One of the most effective ways of its removal from aqueous solution is adsorption on the carbon-based materials surface [17,18].

Therefore, the conducted research was aimed at the synthesis of three types of activated biocarbons and their application in the process of Cu(II) and Pb(II) ions removal from aqueous solutions. Different precursors of activated biocarbons were applied, namely domestic plum stones, pine sawdust and horsetail herb. The activated biocarbons obtained via chemical activation were characterized in terms of their texture, elemental composition and acidic-basic properties. The mechanism of heavy metals ions adsorption was determined on the basis of a collective analysis of the adsorption results (including modeling of isotherms and process kinetics), XPS data, as well as electrokinetic results including determination of surface charge density and zeta potential of the examined solids.

## 2. Materials and Methods

### 2.1. Materials

The precursors of the activated biocarbons were: plum stones (*Prunus domestica*) crushed to a size of 1.0 mm, pine sawdust (*Pinus sylvestris* L.) with a particle size of 5 mm and horsetail herb (*Equisetum arvense*) cut into pieces with a length of 10 mm. All of the materials came from the Wielkopolska region (Poland). For each of the starting materials, a slightly different activation procedure was applied: (1) Plum stones (P) were firstly subjected to pyrolysis process at 500 °C for 60 min under nitrogen atmosphere (flow rate of 10 dm^3^/h). Next, the obtained char was physically mixed with the freshly ground potassium hydroxide (POCh) at the weight ratio of 4:1 and subjected to heat treatment at 700 °C for 30 min under a stream of nitrogen with a flow rate of 20 dm^3^/h. (2) Pine sawdust (S) were at the beginning impregnated with a solution of urea (Chempur) at the weight ratio of 1:1, dried to constant mass at 110 °C and then subjected to pyrolysis step at 700 °C for 60 min (nitrogen flow of 10 dm^3^/h). The char obtained was impregnated with potassium carbonate (POCh) at the weight ratio of 2:1, dried to constant mass at 110 °C and finally subjected to thermal treatment at 700 °C for 45 min, under nitrogen atmosphere (flow rate of 20 dm^3^/h). (3) In turn, the horsetail herb (H) was directly subjected to a chemical activation process, that is, without the pyrolysis stage. The precursor was impregnated with potassium carbonate solution (POCh) at the weight ratio of 2:1, dried to constant mass at 110 °C and subjected to thermal treatment at 800 °C for 30 min, under nitrogen atmosphere (flow rate of 20 dm^3^/h). The products of activation were subjected to two-steps washing procedure, firstly with hot 5% solution of hydrochloric acid (Chempur) and later with hot demineralised water until it became free of chloride ions. The washed activated biocarbons were dried at 110 °C to constant mass and labelled as PAC, SAC and HAC, respectively.

The salts Pb(NO_3_)_2_ (Sigma-Aldrich, St. Louis, MO, USA) and CuSO_4_∙5H_2_O (POCh) were used as a source of metal ions. The stock solutions with a concentration of 1000 ppm were obtained by dissolving these salts in redistilled water using 1 dm^3^ flasks and stored in a fridge. The NaCl solution (POCh) with concentration 0.001 mol/dm^3^ was used as the basic electrolyte. The HCl (Chempur) and NaOH (POCh) solutions with concentrations ranging from 0.01 to 1 mol/dm^3^ were used to the adjustment of the solution pH. The PAR reagent (4-(2-pyridylazo) resorcinol) and ammonium buffer (pH = 10) from Sigma-Aldrich were applied for the determination of Pb(II) ions concentration, whereas in case of Cu(II) ions 25% ammonia solution (Sigma-Aldrich) was used. All reagents were of analytical grade.

### 2.2. Adsorption Experiments

In order to obtain calibration curves, solutions with final concentrations of lead(II) and copper(II) ions changing in the range of 1–200 mg/dm^3^ were prepared. The appropriate volume of the initial Pb(II) or Cu(II) solution and the NaCl basic electrolyte (with initial concentration 0.1 mol/dm^3^) were added to each solution and next they were filled to the required volume with redistilled water.

For the determination of lead(II) concentration in the solution, a spectrophotometric method was used, based on the reaction of Pb(II) cations with 4-(2-pyridylazo) resorcinol (PAR) in an alkaline environment. As a result of this reaction, a stable red chelate complex is formed, the intensity of which is proportional to the content of lead(II) cations in the solution [19]. According to this procedure, 5 cm^3^ of the obtained lead(II) solution was introduced into 25 cm^3^ flask, next 4 cm^3^ of PAR reagent and 10 cm^3^ of ammonium buffer (pH = 10) were added and made up to the mark with redistilled water. Then its absorbance was measured at λ = 520 nm using a Varian UV/VIS Cary 100 spectrophotometer (Palo Alto, CA, USA) coupled with a computer.

The content of copper(II) ions was determined using the reaction between Cu^2+^ cations and ammonia, the product of which is a complex compound [Cu(NH_3_)_4_]^2+^ with an intense blue color [20]. In order to determine these ions concentration, 10 cm^3^ of the obtained Cu(II) solution was placed into 25 cm^3^ flasks and 1 cm^3^ of 25% ammonia solution was added. Such prepared solution was made up to the mark with redistilled water. The absorbance of the samples was measured at λ = 608 nm.

For the determination of adsorbed amounts of heavy metal ions and kinetics of their adsorption the analogous procedures were applied. These experiments were performed at pH 5 at 25 °C in the heavy metal ions concentration changing in the range 1–200 mg/dm^3^ using the following weights of activated biocarbons: 0.012 g of PAC, 0.02 g of SAC and 0.024 g of HAC (per 20 cm^3^ of the solution). The prepared suspensions were shaken in a OLS 200 shaking water bath (Grant Instruments, Cambridge, UK). After 24 h of adsorption, the solids were centrifuged using the MPW 233e type centrifuge (MPW Med. Instruments, Warsaw, Poland) and then the clear filtrates were collected for spectrophotometric analysis. Based on the difference in the initial concentrations of the examined ions and their concentrations after the adsorption process, their adsorbed amounts were calculated:

(1) $$q_{e}=\frac{\left(C_{0}-{\;C}_{e}\right)}{m}\cdot \;V$$

where: C_0_ and C_e_—heavy metal ions concentrations in the solution before adsorption and at the equilibrium state, respectively [mg/dm^3^], V—volume of the solution [dm^3^], m—mass of the solid [g].

For the modeling of adsorption isotherms [21,22], the Langmuir and Freundlich equations were used:

(2) $$q_{e}=\frac{q_{m}K_{L}C_{e}}{1+K_{L}C_{e}}$$

(3) $$q_{e}=K_{F}C_{e}^{1/n}$$

where: q_e_—equilibrium adsorbed amount [mg/g], q_m_—maximal adsorbed amount (monolayer capacity) [mg/g], K_L_—Langmuir constant [dm^3^/mg], C_e_—equilibrium concentration of metal ions [mg/dm^3^], K_F_—Freundlich constant [mg/g (mg/dm^3^)^1/n^], n—Freundlich parameter.

The adsorption kinetics of Pb(II) and Cu(II) ions on the surface of activated biocarbons was determined at pH 5 using the measurement procedure described above. The spectrophotometric measurements of the examined systems were performed at specific time intervals—after 5, 10, 15, 30, 60, 90, 120, 180 min. The starting concentration of both metals ions was 200 mg/dm^3^.

Two models of adsorption kinetics [23] were selected: the pseudo-first-order proposed by Lagergren and the pseudo-second-order proposed by Ho and McKay, represented by the equations:

(4) $$\frac{{\mathrm{dq}}_{t}}{\mathrm{dt}}=k_{1}\left(q_{e}-q_{t}\right)$$

(5) $$\frac{{\mathrm{dq}}_{t}}{\mathrm{dt}}=k_{2}{\left(q_{e}-q_{t}\right)}^{2}$$

where: q_t_—amount of metal ions adsorbed after time t [mg/g], k_1_ [1/min] and k_2_ [g/mg∙min]—equilibrium rate constants [mg/g].

The possibility of activated biocarbons regeneration was investigated on the example of more toxic lead(II) ions, using HCl and NaOH solutions with concentration 0.1 mol/dm^3^.

### 2.3. Analytical Procedures

The elemental analysis (C, H, N, S) of the starting plum stones, pine sawdust, horsetail herb as well as each activated biocarbon samples was performed using a Vario EL III elemental analyzer (Elementar Analysensysteme GmbH, Langenselbold, Germany). The total mineral matter (ash) content was determined by burning the carbonaceous materials in a microwave muffle furnace (Phoenix, CEM Corporation, Matthews, IL, USA) at temperature of 815 °C for 60 min, according to the DNS ISO 1171:2002.

The textural characterization of the activated biocarbons was based on nitrogen adsorption-desorption isotherms measured at −196 °C on sorptometer ASAP 2020 manufactured by Micrometrics Instrument Corporation (Norcross, GA, USA). Prior to the isotherm measurements, the carbonaceous samples were out-gassed at 300 °C for 12 h, in order to remove all pre-adsorbed species. BET specific surface area (S_BET_) was evaluated in the range of relative pressures p/p_0_ of 0.05–0.30. Total pore volume (V_t_) was calculated by converting the amount adsorbed at p/p_0_~0.99 to the volume of liquid adsorbate. Average pore diameter was calculated (D) from equation D = 4V_t_/S_BET_. The commonly known t-plot method was applied to determine the micropore volume and area. Pore size distribution was estimated by the Density Functional Theory (Model: N_2_ @ 77K on carbon, slit pores, method: non-negative regularization; no smoothing).

The content of the surface functional groups of acidic and basic nature was evaluated according to the Boehm method, described in detail in our earlier paper [24]. Volumetric standards of 0.1 mol/dm^3^ NaOH (POCh) and 0.1 mol/dm^3^ HCl (Chempur) were used as the titrants.

The pH of each activated biocarbon sample was determined according to the following procedure: a portion of 0.5 g of each carbonaceous material in a powder form was mixed with 25 cm^3^ of distilled water and then the suspension was magnetically stirred overnight to reach equilibrium. After that the pH of the suspension was measured (upon continuous stirring) using a CP-401 pH-meter (ELMETRON, Zabrze, Poland) equipped with an EPS-1 combination glass electrode, calibrated with standards solutions of pH 3, 7 and 10.

The morphology of the activated biocarbons was analyzed using high-resolution environmental scanning electron microscope Quanta 250 FEG provided by FEI Company (Hillsboro, OR, USA).

The XPS (X-ray photoelectron spectroscopy) apparatus (Gammadata Scienta, Uppsala, Sweden) was used to determine elemental composition of the solids and adsorbed forms of heavy metals.

### 2.4. Electrokinetic Measurements

Potentiometric titration was carried out using a set consisting of the following devices and elements: water-thermostated Teflon vessel, thermostat (Lauda RE 204, Delran, NJ, USA), electrodes: glass and calomel (Beckman Instruments, Brea, CA, USA), pH-meter PHM 240 (Radiometer, Copenhagen, Denmark), laboratory stirrer, automatic micro-burette Dosimat 765 (Methrom, Herisau, Switzerland), computer with Titr_v3 software authored by W. Janusz [25]. Firstly, 50 cm^3^ of the basic electrolyte solution was introduced into a Teflon vessel. An appropriate amount of HCl solution (0.01 moL/cm^3^) was added to obtain an initial pH in the range of 3–3.5. The system was titrated with NaOH solution with concentration 0.1 mol/dm^3^. In this way, a reference curve was obtained—it shows the relationship between the solution pH and the volume of added base. Then, in an analogous manner, the potentiometric titration of the suspensions containing activated biocarbon was carried out with and without the addition of Pb(II) or Cu(II) cations (with concentrations of 10 mg/dm^3^). The weights of adsorbents used were as follows: 0.007 g of PAC, 0.02 g of SAC and HAC. The solid surface charge (σ_0_, [μC/cm^2^]) was calculated from the formula:

(6) $$\sigma_{0}=\frac{\Delta {\mathrm{VC}}_{b}F}{\mathrm{mS}}\;$$

where: C_b_—base (NaOH) concentration [mol/dm^3^], F—Faraday constant [C/mol], m—solid mass in the suspension (g), S—specific surface area of the solid [m^2^/g], ΔV—difference in the volume of base which must be added to obtain the pH of suspension and reference solution (basic electrolyte) to the specified value [dm^3^].

The Zetasizer Nano ZS (Malvern Instruments Ltd., Malvern, UK) was used to measure the electrophoretic mobility (U_e_, [m^2^/Vs]) of examined suspensions, applying the immersion cell. Before measurements, the tested suspensions were subjected to ultrasounds using sonicator XL 2020 (Beckman Instruments).

The Henry equation [26] was used to obtain the zeta potential (*ζ*, [mV]) value:

(7) $$U_{e}=\frac{2\varepsilon_{0}\varepsilon \zeta }{3\eta }f\left(\kappa a\right)$$

where: ε—dielectric constant of liquid medium, ε_0_ is—electric permeability of vacuum [F/m], η—viscosity of liquid medium [Pa⋅s], f(κa)—the Henry function (a—solid particle radius [nm], 1/κ—thickness of the electric double layer [nm]).

The activated biocarbon suspensions were prepared by mixing the appropriate volumes of the basic electrolyte and heavy metal ions to ensure their concentrations of 0.001 mol/dm^3^ and 10 mg/dm^3^ (per 100 cm^3^ of the solution), respectively, and adding the appropriate portions of activated carbons (0.013 g of PAC, 0.02 g of SAC and HAC). Each suspension was sonicated for three minutes and divided into five portions. Then, in each of them, the appropriate pH value was adjusted, varying by one unit in the range from 3 to 7. The samples were placed in a quartz cuvette and measured manually using a dip cell. Before each measurement, the cell was rinsed twice with the examined suspension.

## 3. Results

### 3.1. Elemental Composition of the Precursors and Activated Biocarbons Prepared

According to the data presented in Table 1, each of the used starting materials contains about 45% wt. of elemental carbon in the structure, which indicates their potential suitability for the production of activated biocarbons. Importantly, two of them, i.e., plum stones and pine sawdust, are characterized by a very low content of mineral admixtures, which is a desirable feature as mineral matter adversely affects the physicochemical and sorption parameters of the activation products—it is just an unnecessary ballast. The precursors used for the research differ also significantly in terms of the content of heteroatoms, such as nitrogen, sulphur and oxygen. The greatest contribution of non-carbon admixtures (as in case of the ash) is found in the structure of the horsetail herb.

As follows from the further analysis of the data presented in Table 1, chemical activation process (irrespective on the procedure) caused significant changes in the structure of the initial materials. First of all, samples PAC, SAC and HAC show more than twice the content of elemental carbon than the corresponding precursors. With increasing contribution of C^daf,^ a considerable decrease in the contents of hydrogen, nitrogen, sulphur and especially oxygen is noted in the activated biocarbons obtained. These changes are induced by the acting of very reactive activating agents—KOH or K_2_CO_3_ as well as the high temperature of the activation process, as a result of which the least stable fragments of the carbonaceous structure are decomposed. Thermochemical treatment of the waste biomass leads also to an increase in the contribution of mineral admixtures in the structure of activation products.

### 3.2. Acidic-Basic Properties of the Activated Biocarbons Prepared from Waste Biomass

In order to establish the chemical nature of surface of the activated biocarbons prepared, the pH value of water extracts was measured and the content of surface functional groups of acidic and basic character was determined. The data presented in Table 2 imply that the biocarbons studied show quite diverse acidic-basic properties, which is evidenced by the content of surface functional species ranging from 1.13 to 1.94 mmol per gram and pH value varying from 6.52 to 7.66. The greatest amount of the functional groups was found on the surface of sample PAC obtained via chemical activation of plum stones with potassium hydroxide, whereas the least functionalized surface is found in the sample SAC obtained from pine sawdust. According to the data from Table 2, the activated biocarbons prepared differ also significantly in terms of the content of acidic and basic groups. The PAC and HAC samples obtained by activation of plum stones and horsetail herb show the prevalence of acidic groups—they contain almost 2 times more acidic groups than basic ones. The distinct situation is observed in case of SAC sample obtained via nitrogen enrichment and chemical activation of sawdust by potassium carbonate, which exhibits a slight predominance of basic functional species. This difference is probably the result of nitrogen functional groups incorporation into the carbon matrix during the pyrolysis step.

### 3.3. Textural Parameters and Morphology of the Activated Biocarbons Prepared from Waste Biomass

**Figure 1:**
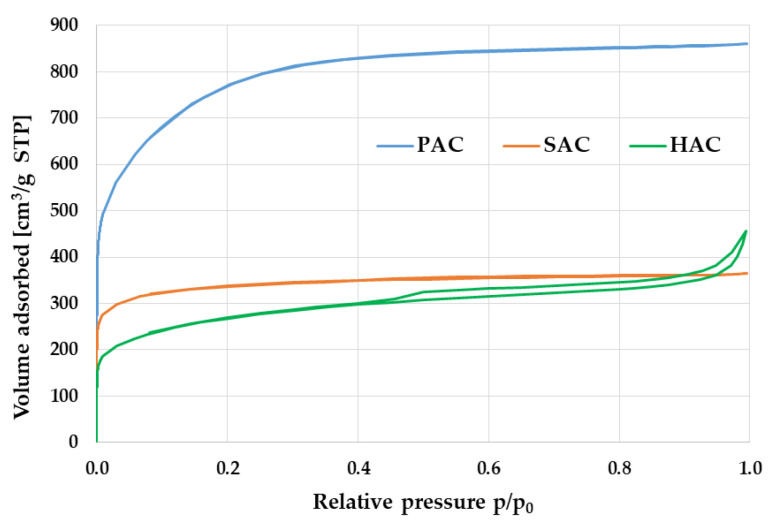
Low-temperature N_2_ adsorption-desorption isotherms of PAC, SAC and HAC activated biocarbons.

**Figure 2:**
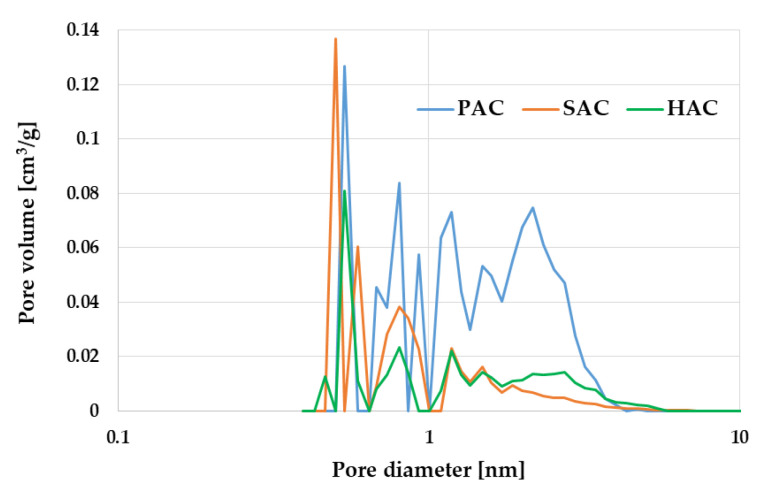
Pore size distribution of PAC, SAC and HAC activated biocarbons.

The potential application of carbonaceous adsorbents is determined by the degree of their surface area development and the type of their porous structure. According to the data presented in Table 3 and in Figure 1 and Figure 2, all the samples obtained have well-developed surface area and porous structure with dominant of micropores. Textural parameters of the activated biocarbon samples depend significantly on the type of precursor used and the procedure of its thermochemical processing. The most developed surface area (2759 m^2^/g) was found in the PAC sample activated by KOH, which is the consequence of its high reactivity as an activating agent. In case of samples activated with potassium carbonate (HAC and SAC) the specific surface area is much smaller and varies in the range from 955 m^2^/g to 1266 m^2^/g. A highly probable reason for the less favourable textural parameters of the HAC sample is a high content of mineral matter in the precursor structure (Table 1), that hinders the access of activator to the carbon matrix upon direct activation and thus prevents the effective development of the porous structure and surface area. Moreover, the mineral substance can act as a ballast filling the porous structure of the final products and causing poorer results of low-temperature nitrogen adsorption/desorption.

**Figure 3:**
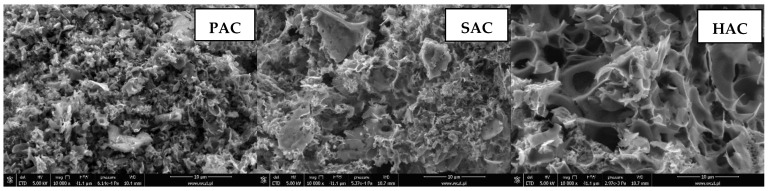
SEM images of PAC, SAC and HAC activated biocarbons.

Heat treatment of the waste biomass in the presence of potassium hydroxide or carbonate leads to materials with a micro-mesoporous structure, which is indicated by a low value of average pore diameter (Table 3) as well as pore size distribution presented in Figure 3. The highest contribution of micropores of 84% is found in PAC sample obtained by chemical activation of plum stones. Significant contribution of micropores in the porous structure of all activated biocarbons under investigation is also confirmed by the course of the low-temperature nitrogen adsorption/desorption isotherms presented in Figure 2. According to the IUPAC organization classification, these isotherms are close to the type I, characteristic of microporous and mesoporous materials with the pore size close to the micropores range. In case of HAC sample, a clear hysteresis loop is observed, the presence of which indicates a greater contribution of mesopores in the porous structure. It is similar to the H4 type loop, which is characteristic of materials containing narrow slit-like pores.

Textural and morphological diversity of the prepared activated biocarbons is con-firmed by SEM photomicrographs presented in Figure 3. As can be seen, depending on the precursor type and the preparation procedure applied the samples differ significantly in terms of the shape, size as well as number and arrangement of pores and slits. The SEM images confirm also that the PAC sample obtained as a result of chemical activation of plum stones with KOH has the most developed porous structure among the tested carbonaceous materials. Slightly brighter fragments visible on each of the micrographs may be a consequence of the mineral matter presence in the structure of activated biocarbons prepared.

### 3.4. Adsorption of Cu(II) and Pb(II) Ions on the Activated Biocarbons Surface

**Figure 4:**
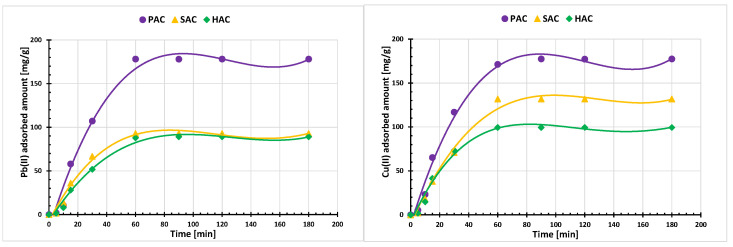
Adsorption kinetics of Pb(II) and Cu(II) ions on the examined solids surface; C_0_ = 200 mg/dm^3^, pH 5.

Figure 4 presents the kinetic results of heavy metal ions adsorption on the activated biocarbons surface. Their analysis clearly shows that after approximately one hour, the system reaches equilibrium and the amount of adsorbed ions remains constant. The parameters of adsorption kinetics modeling presented in Table 4 indicate that a better fit is observed when the pseudo-second-order model is used (the obtained R^2^ coefficients for all tested systems exceed the value of 0.98). This indicates that the binding process of the examined heavy metal ions is primarily the result of their chemisorption on the solid surface. It consists in the sharing or exchange of electrons between ions and active surface groups of the adsorbent [27].

**Figure 5:**
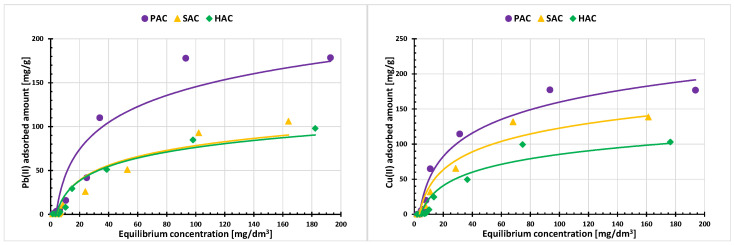
Adsorption isotherms of Pb(II) and Cu(II) ions on the examined solids.

**Figure 6:**
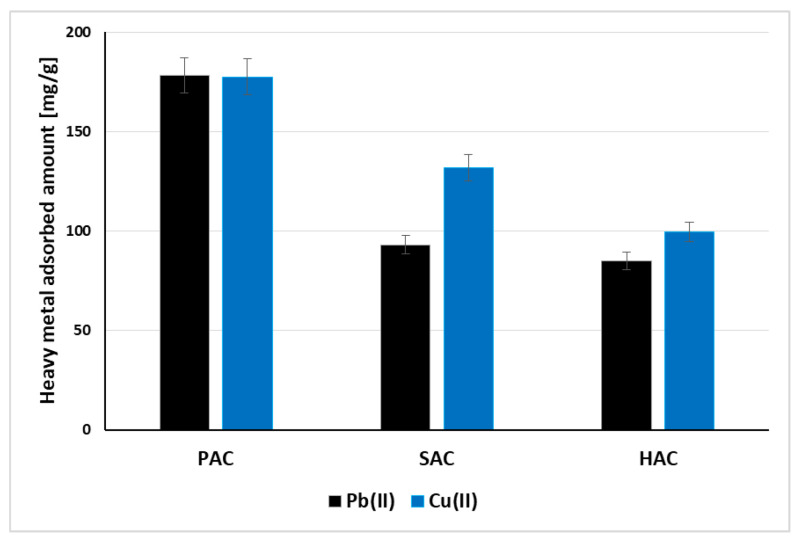
Comparison of adsorbed amounts of Pb(II) and Cu(II) ions on the examined solids surface; C_0_ 200 mg/dm^3^, pH 5.

The adsorption isotherms of Pb(II) and Cu(II) ions on the surface of three examined activated biocarbons are shown in Figure 5, whereas Figure 6 presents a comparison of the adsorbed amounts of both ions. A similar dependence of the amount of the adsorbed metal cations on the size of the specific surface area of the samples is observed. The highest adsorption of Pb(II) and Cu(II) occurs on the surface of the PAC activated biocarbon with the most developed specific surface area (2759 m^2^/g), and the lowest in case of HAC sample, whose S_BET_ is only 955 m^2^/g. The larger the specific surface area and the greater participation of micropores in the adsorbent structure, the greater the amount of heavy metal ions can penetrate into these spaces and can be adsorbed. The best fit of the adsorption data was obtained for the Langmuir model (Table 5). This indicates that the lead(II) and copper(II) ions interacting with the solid surface form an energetically homogeneous monolayer [28].

Lead and copper cations adsorb in similar amounts on the surfaces of PAC and HAC activated carbons at pH 5 (Figure 6). A significant difference is visible in case of SAC solid, which is characterized by the smallest pore size-copper(II) adsorption is about 40 mg/g higher than that of lead(II). It can be the result of different metal species presented in the solution within the acidic range of pH values and their different size. In the solutions of pH lower than 6, the dominant species of copper are Cu^2+^ cations [29], whereas in case of lead the hydrated forms (mainly PbOH^+^) also exists besides Pb^2+^ ones. Due to the hydration of Pb(II) its diameter increases, which limits their penetration into the adsorbent pores. As a consequence, the adsorption of Pb(II) ions on the SAC surface is noticeably lower than Cu(II) ions.

The hydrochloric acid proved to be more effective desorbing agent of Pb(II) ions than sodium base. In case of its usage, the lead desorption was significantly greater and ranged from about 65% for PAC sample to 80% for SAC and HAC activated biocarbons. In turn, NaOH caused the Pb(II) desorption on the level ranging from about 12% in case of PAC to 20% for the SAC and HAC samples.

**Figure 7:**
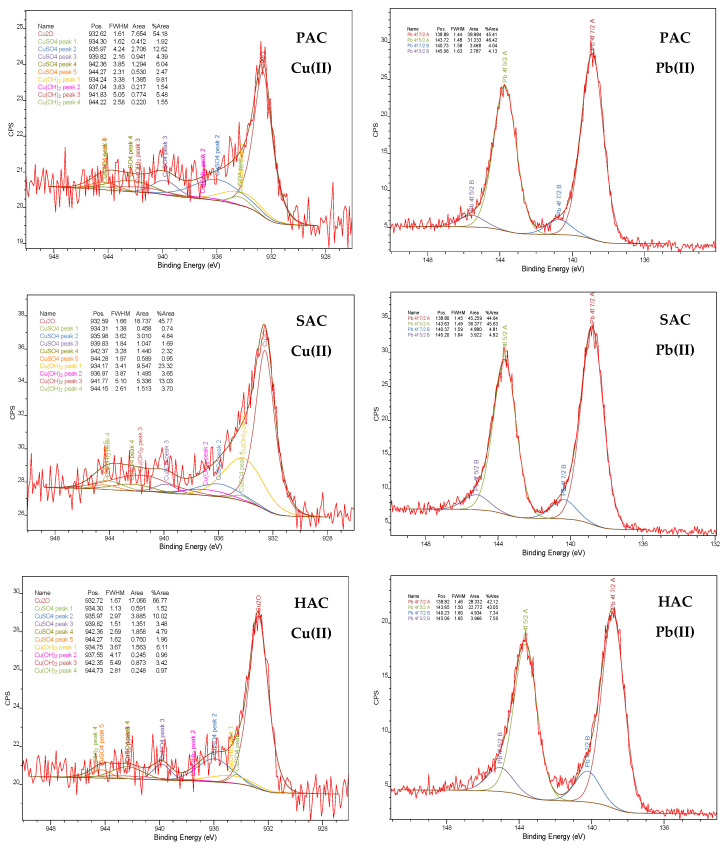
The Cu 2p and Pb 4f spectra of activated biocarbons after heavy metal ions adsorption.

The XPS spectra of PAC, SAC and HAC activated biocarbons obtained after heavy metal ions adsorption (Figure 7 and Table 6) confirmed the presence of copper and lead ions on the solids surface. Moreover, based on the analysis of Cu 2p spectrum (Table 7) it can be stated that in solutions of pH close to 5 the dominant copper species are Cu^+1^ and Cu^2+^ ions, so its adsorption is governed mainly by ionic exchange. As far as lead species are concerned (Table 8), a comparable amount of are Pb^2+^ cations and Pb(OH)_2_ are observed, so it can be stated that a significant part of lead is precipitated as hydroxides on the carbonaceous adsorbents surface.

Table 9 contains the comparison of Pb(II) and Cu(II) adsorbed amounts on the different carbonaceous adsorbents described in the literature and in the present manuscript. According to these data, the majority of the materials adsorb much more lead ions than copper ones. However, it should be emphasized that maximum sorption capacity of PAC sample towards Pb(II) as well as Cu(II) ions is much higher than for the other carbonaceous adsorbents. This is especially pronounced in case of copper ions removal from aqueous solutions.

### 3.5. Electrokinetic Properties of the Activated Biocarbons

**Figure 8:**
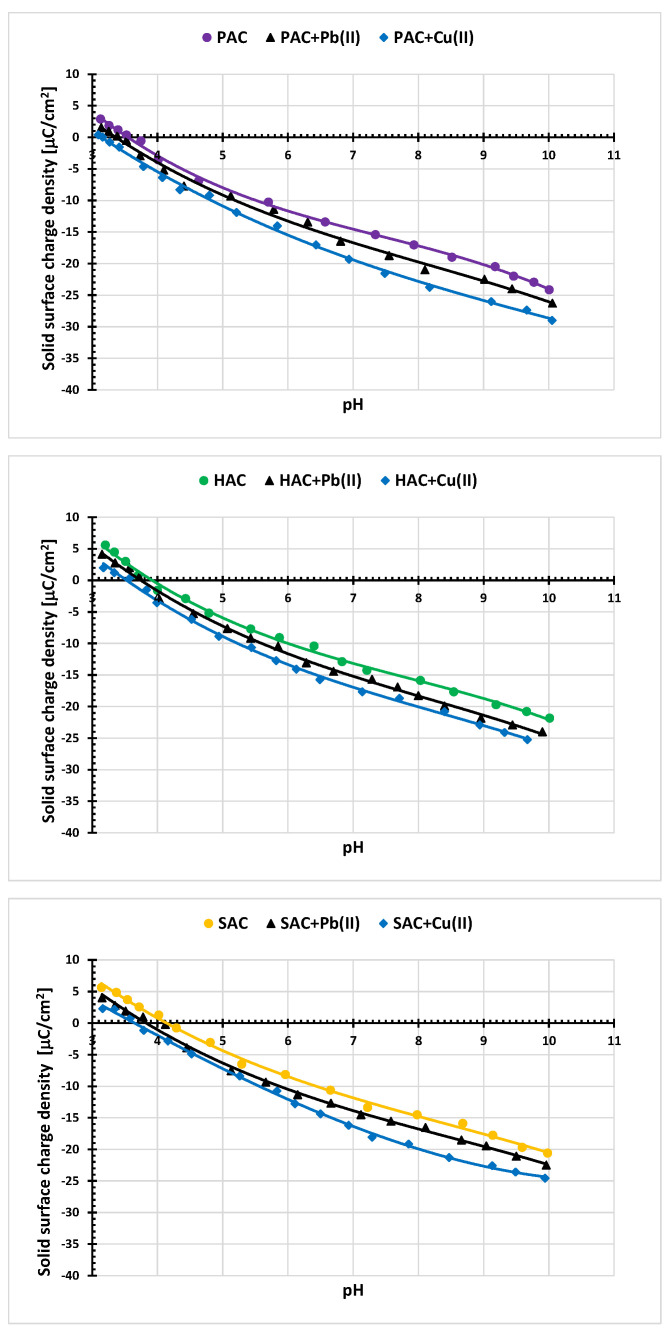
Surface charge density of PAC, SAC and HAC samples without and in the presence of heavy metals ions; C_0_ 10 mg/dm^3^.

Figure 8 shows changes in the surface charge density of the examined carbon materials without and in the presence of Pb(II) or Cu(II) ions as a function of solution pH. Analysis of these data indicates that the point of zero charge (pzc) for all tested activated biocarbons is in the range of pH 3.6–4.1. For pH above pH_pzc_ value, the adsorbent surface is negatively charged, and below-positively. Thus, at pH 5 (at which the ions adsorption process was carried out), there are favorable electrostatic conditions for metal cations binding to the negatively charged surface of the adsorbent. For all three activated biocarbons in the entire tested pH range, the decrease in the surface charge density is observed in the heavy metal ions presence, compared to the system without these ions. A slightly greater reduction is observed in case of the copper(II) ions addition. This behavior is typical of simple inorganic ions. Adsorption of small cations causes the formation of additional groups with a negative charge on the surface of a solid, which is manifested by a decrease in the density of the surface charge [36]. Moreover, the smaller size of Cu(II) ions makes that this phenomenon is more pronounced in case of these cations.

**Figure 9:**
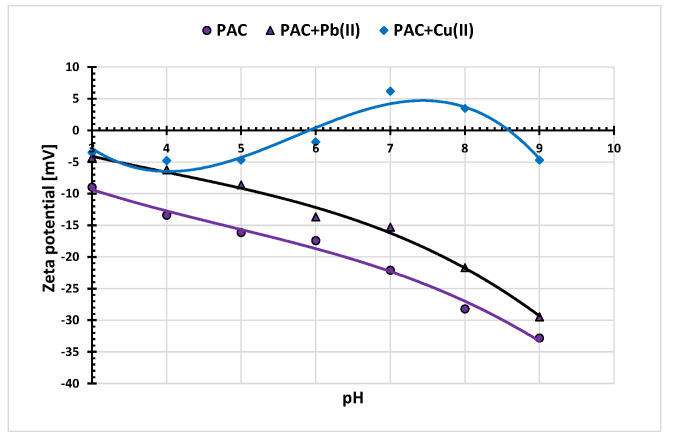

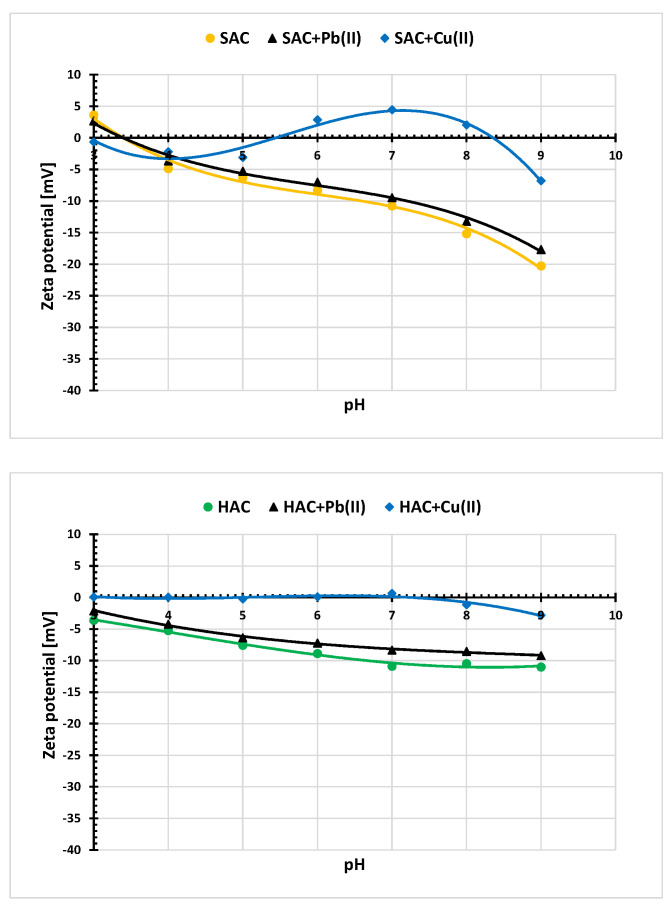
Zeta potential of PAC, SAC and HAC solid particles without and in the presence of heavy metals ions; C_0_ 10 mg/dm^3^.

In the tested pH range, the zeta potential of PAC and HAC activated biocarbons in the absence of heavy metal ions assumes only negative values (Figure 9). The isoelectric point (iep) is only observed for SAC sample at pH of 3.3. This means that for this pH value the total charge of the slipping plane is zero. For each tested solid, the presence of adsorbed metal ions causes increase in the zeta potential. Its highest values are observed in the presence of Cu(II) ions. Changes in the zeta potential of solid particles in the presence of metal cations result mainly from the change in the ionic composition of the slipping plane area due to the removal of the basic electrolyte ions by the adsorbing Pb(II) and Cu(II) ions. This mainly concerns the Na+ ions, which lead to an increase in the electrokinetic potential of the solid aqueous suspension. Moreover, in case of Cu(II) cations the increase in zeta potential in the pH range 6–8 (maximum on the curves) for PAC and SAC systems is observed. This is due to the effect of overcharging (or overloading) of the electrical double layer (edl), i.e., charge reversal. This results from the occurrence of more numerous cations on the inner part of edl than their number on the solid surface. Consequently, the same charge sign appears on the edl outside part and also on the surface [37].

## 4. Conclusions

Waste plum stones, pine sawdust and horsetail herb were used as the precursors of new and effective carbonaceous adsorbents. The chemical activation of such kind of biomass leads to micro-mesoporous activated biocarbons, characterized by diverse elemental composition, acidic-basic character of the surface as well as sorption abilities towards lead and copper ions. The highest adsorption of both heavy metals ions occurs on the surface of the plum stones-based activated biocarbon with the most developed specific surface area (2759 m^2^/g)—it reaches a level of about 180 mg/g. The modelling of adsorption data indicated that lead(II) and copper(II) ions react with the solid surface mainly through chemical interactions and form an energetically homogeneous monolayer. A significant difference in metal adsorbed amounts is visible in case of SAC activated biocarbon characterized by the smallest pore size, for which Cu(II) is adsorbed in greater amounts. It can be the result of different metal forms and their sizes present in the solution at pH 5, which affects their ability to penetrate into porous structure of the solids. The XPS analysis showed that the main forms of heavy metal ions in adsorption layer are: PbO/Pb_3_O_4_ and Pb(OH)_2_ in case of lead as well as Cu_2_O, CuSO_4_ and CuO/Cu(OH)_2_ for copper.

## Figures and Tables

**Table 1:** Elemental composition of the precursors and activated biocarbons prepared as well as the yield of the chemical activation process (wt.%).

Sample	Ash	C^daf 1^	H^daf^	N^daf^	S^daf^	O^diff 2^	Yield
P	0.5	46.1	5.4	0.1	0.1	48.3	-
S	0.6	46.6	7.0	1.3	1.0	44.1	-
H	6.8	44.9	5.6	3.0	1.5	45.0	-
PAC	1.2	92.3	0.7	0.1	0.0	6.9	57
SAC	2.3	93.0	1.0	0.8	0.3	4.9	49
HAC	8.7	90.5	2.1	1.4	0.0	6.0	41

^1^ dry-ash-free basis; ^2^ calculated by difference.

**Table 2:** Acidic-basic properties of the activated biocarbons obtained.

Sample	pH	Basic Groups Content [mmol/g]	Acidic Groups Content [mmol/g]	Total Content of Surface Groups [mmol/g]
PAC	6.52	0.65	1.29	1.94
SAC	6.88	0.67	0.46	1.13
HAC	7.66	0.54	1.01	1.55

**Table 3:** Textural parameters of the activated biocarbons obtained.

Sample	Surface Area [m^2^/g]	Total Pore Volume [cm^3^/g]	Micropore Contribution [%]	Average Pore Diameter [nm]
PAC	2759	1.332	84	1.931
SAC	1266	0.565	82	1.787
HAC	955	0.705	61	2.954

**Table 4:** Kinetic parameters obtained for modelling of heavy metal ions adsorption on the examined solids surface; C_0_ = 200 mg/dm^3^ ppm, pH 5.

Adsorbed Ions	Kinetic Parameters of Pseudo-First Order Model								
	q_e_ [mg/g]			k_1_ [1/Min]			R^2^		
	PAC	SAC	HAC	PAC	SAC	HAC	PAC	SAC	HAC
Pb(II)	96.60	44.60	5.58	0.1199	0.1068	0.0308	0.7136	0.707	0.1664
Cu(II)	156.05	76.24	46.17	0.1128	0.1202	0.1107	0.7300	0.7292	0.7067
**Adsorbed Ions**	**Kinetic Parameters of Pseudo-Second Order Model**								
	**q_e_ [mg/g]**			**k_2_ [g/(mg∙min)]**			**R^2^**		
	**PAC**	**SAC**	**HAC**	**PAC**	**SAC**	**HAC**	**PAC**	**SAC**	**HAC**
Pb(II)	166.66	105.26	100.00	0.00045	0.00053	0.00014	0.9877	0.9847	0.9807
Cu(II)	181.81	144.92	111.11	0.00235	0.00035	0.00058	0.9999	0.9857	0.9883

**Table 5:** Adsorption parameters obtained for modelling of heavy metal ions isotherms on the examined solids surface; pH 5.

Adsorbed Ions	Adsorption Parameters of Langmuir Model								
	qm [mg/g]			KL [dm^3^/mg]			R^2^		
	PAC	SAC	HAC	PAC	SAC	HAC	PAC	SAC	HAC
Pb(II)	235.77	231.25	174.65	0.01996	0.00123	0.00123	0.991	0.970	0.997
Cu(II)	198.83	183.91	160.94	0.00033	0.00114	0.00115	0.994	0.965	0.993
**Adsorbed Ions**	**Adsorption Parameters of Freundlich Model**								
	**n**			**K_F_ [mg/g·(mg/dm^3^)^1/n^]**			**R^2^**		
	**PAC**	**SAC**	**HAC**	**PAC**	**SAC**	**HAC**	**PAC**	**SAC**	**HAC**
Pb(II)	0.678	0.669	0.606	0.00309	0.00713	0.00529	0.904	0.836	0.821
Cu(II)	0.553	0.617	0.518	0.00177	0.00221	0.00275	0.643	0.829	0.880

**Table 6:** Elemental composition of the activated biocarbons surface after adsorption of heavy metal ions [at. %].

Sample	C	N	O	S	Si	Cu	Pb
PAC + Cu(II)	90.4	0.0	7.2	1.2	0.7	0.5	-
SAC + Cu(II)	89.1	1.5	8.1	0.4	0.6	0.3	-
HAC + Cu(II)	80.5	1.6	12.6	1.3	3.5	0.5	-
PAC + Pb(II)	91.8	0.4	7.2	0.0	0.0	-	0.6
SAC + Pb(II)	92.6	0.9	5.5	0.2	0.3	-	0.5
HAC + Pb(II)	85.0	1.3	10.3	0.0	2.8	-	0.6

**Table 7:** The main Cu 2p photopeaks of PAC, SAC and HAC activated biocarbons.

Sample	Species	Binding Energy [eV]	Relative Content [%]
PAC	Cu_2_O	932.72	66.8
	CuSO_4_	935.97	10.0
	CuO or Cu(OH)_2_	934.75	6.1
SAC	Cu_2_O	932.62	54.2
	CuSO_4_	935.97	12.6
	CuO or Cu(OH)_2_	934.24	9.8
HAC	Cu_2_O	932.59	45.8
	CuSO_4_	935.98	4.8
	CuO or Cu(OH)_2_	934.17	23.3

**Table 8:** The Pb 4f photopeaks of PAC, SAC and HAC activated biocarbons.

Sample	Species	Binding Energy [eV]	Relative Content [%]
PAC	PbO or Pb_3_O_4_	138.89	42.1
	Pb(OH)_2_	143.72	43.1
	PbSO_4_	140.73	7.3
	Pb(NO_3_)_2_	145.56	7.5
SAC	PbO or Pb_3_O_4_	138.82	42.1
	Pb(OH)_2_	143.65	43.1
	PbSO_4_	140.23	7.3
	Pb(NO_3_)_2_	145.06	7.5
HAC	PbO or Pb_3_O_4_	138.80	44.6
	Pb(OH)_2_	143.63	45.6
	PbSO_4_	140.37	4.8
	Pb(NO_3_)_2_	145.20	4.9

**Table 9:** Adsorption capacity toward Pb(II) and Cu(II) ions for various carbonaceous adsorbents.

Adsorbent	Maximum Adsorbed Amount [mg/g]	Reference
PAC	Pb(II) 178.1 mg/g Cu(II) 177.5 mg/g	[this study]
Activated carbon obtained from *Eucalyptus camaldulensis Dehn* bark	Pb(II) 110.6 mg/g Cu(II) 28.9 mg/g	[30]
Granular activated carbon derived from coconut shells	Pb(II) 10.8 mg/g Cu(II) 3.6 mg/g	[31]
Peat	Pb(II) 118.7 mg/g Cu(II) 34.0 mg/g	[32]
Chitosan-pyromellitic dianhydride modified rice straw biochar	Pb(II) 13.9 mg/g Cu(II) 96.1 mg/g	[33]
Date seed biochar	Pb(II) 148.8 mg/g Cu(II) 26.7 mg/g	[34]
Pepper stem biochar	Pb(II)~120 mg/g Cu(II)~45 mg/g	[35]

## Data Availability

Data are contained within the article.
